# Supplementary material for: Effects of Upper Body Exercise Training on Aerobic Fitness and Performance in Healthy People: A Systematic Review
Source: Biology (Basel). 2023 Feb 23;12(3):355. doi: 10.3390/biology12030355 (PMC10045299; doi:10.3390/biology12030355)
Supplement: Supplementary file 1 [file biology-12-00355-s001.zip › biology-2170722-supplementary.pdf]

Table S1. Search terms used for PubMed, Web of Science, Scopus and EMBASE

Database	Category		
	Training tool	Training intervention	Outcomes
PubMed	upper body [Title/Abstract] upper limb [Title/Abstract] handcycl* [Title/Abstract] armcycl*[Title/Abstract] hand crank* [Title/Abstract] arm crank* [Title/Abstract] arm ergomet* [Title/Abstract] small muscle [Title/Abstract] upper body [Title/Abstract] wheelchair treadmill [Title/Abstract] wheelchair ergomet* [Title/Abstract]	train* [Title/Abstract] exercis* [Title/Abstract] interven*[Title/Abstract] speed interval [Title/Abstract] SIT [Title/Abstract] LIT [Title/Abstract] HIT [Title/Abstract] HIIT [Title/Abstract] high intensity [Title/Abstract]	VO2 [Title/Abstract] VO2peak [Title/Abstract] VO2max [Title/Abstract] Power [Title/Abstract] Oxygen uptake [Title/Abstract] oxygen consumption [Title/Abstract] cardiopulmonary [Title/Abstract] fitness [Title/Abstract]
Web of Science	upper body upper limb handcycl* armcycl* hand crank* arm crank* arm ergomet* small muscle upper body wheelchair treadmill wheelchair ergomet* (Topic)	train* exercis* interven* speed interval SIT LIT HIT HIIT high intensity (Topic)	VO2 VO2peak VO2max Power Oxygen uptake oxygen consumption cardiopulmonary fitness (Topic)
Scopus	TITLE-ABS-KEY (upper body upper limb handcycl* armcycl* hand crank* arm crank arm ergomet* small muscle upper body wheelchair treadmill wheelchair ergomet*)	TITLE-ABS-KEY (train* exercis* interven* speed interval SIT LIT HIT HIIT high intensity)	TITLE-ABS-KEY (VO2 VO2peak VO2max Power Oxygen uptake oxygen consumption cardiopulmonary fitness)
EMBASE	(upper limb handcycl* arm cycl* arm crank* arm ergomet* small muscle upper body wheelchair treadmill wheelchair ergomet*):ti,ab,kw.	(train* exercis* interven* speed interval SIT LIT HIT HIIT high intensity):ti,ab,kw.	(VO2 VO2peak VO2max power oxygen uptake oxygen consumption cardiopulmonary fitness):tb,ab,kw.

Table S2. Test devices

Study		Test device
**Abonie 2021**	**H**	Instrumented handcycle mounted on a motor driven treadmill (Enraf Nonius, The Netherlands)
**Bhambani 1991**	**A**	Arm cycling (Monark Rehab Trainer, Model 881)
**De Groot 2008**	**W**	Standardized wheelchairs (VU: Quickie triumph, Reade: Sopur starlight) on motor driven treadmill (Enrad Nonius, The Netherlands)
**De Groot 2013**	**W**	Standardized wheelchairs (VU: Quickie triumph, Reade: Sopur starlight) on motor driven treadmill (Enrad Nonius, The Netherlands)
**El Sayed 2003**	**A**	Arm cranking ergometer
**Glaser 1981**	**W**	Wheelchair ergometer (basically an extension of the popular Monark bicycle ergomter)
**Goosey-Tolfrey 2011**	**W**	Basketball wheelchair (Quattro, RGK, Burntwood, Staffordshire, England)
**Grange 2002**	**W**	Classic wheelchair (seat dimensions 15 inches by 18 inches, seat height 30 inches)
**Hettinga 2016**	**H**	Attach-unit handcycle (consisting of a handrim wheelchair connected with a mounted handcycling unit) on a motor driven treadmill (Enrad Nonius, The Netherlands)
**Hill 2018**	**A**	Arm cranking ergometer (Lode Angio BV, Groningen, Netherlands)
**La Monica 2019**	**A**	Modified cycle ergometer (894E, Monark Cycle Ergometer, Vansbro, Sweden) that was affixed to adjustable scaffolding for arm cranking
**Lewis 1980**	**A**	Collins ergometer (model PE) which had been modified to accommodate both arm cranking and leg cycling.
**Magel 1978**	**A**	Standard bicycle ergometer (Monark) was adapted for arm work by removing the rear assembly
**McKenzie 1978**	**A**	Bicycle ergometer; arm-trained group pedalled the ergometer while seated with the legs forward and supported comfortably off the ground (in this manner, the leg muscles could not be used as stabilizers during the arm pedaling movement
**Pinto 2017**	**A**	Arm-cranking device known as the Krankcycle (Matrix, Cottage Grove, WI, USA)
**Pogliaghi 2006**	**A**	Arm cranking ergometer (ARM test, Top-XT, Technogym, Italy)
**Schoenmakers 2016**	**H**	Wheelchair with a mounted handcycling unit (Double Performance, The Netherlands) on a motor driven treadmill (Enraf Nonius, The Netherlands)
**Rasmussen 1975**	**A**	Arm training/leg training (not described)
**Sedlock 1988**	**A**	The Arm crank ergometer (Monark) was an adaptation of a cycle ergometer
**Simmons 1971**	**A**	Fleisch "ergostat" (Jacquet Ltd., Basle, Switzerland) and operating the short arm cranks
**Tordi 2001**	**W**	Wheelchair ergometer consisted of chair wheels connected to standard friction-loaded Fleisch ergometer by means of chain and chainring system
**Loftin 1988**	**A**	A friction resistance Monark bicycle ergometer modified for arm cranking
**Van der Woude 1999**	**W**	Wheelchair ergometer on a motor driven treadmill (Enraf Nonius, model 3446, Delft, The Netherlands)
**Van den Berg 2010**	**W**	Wheelchair (Sopur starlight) on a motor driven treadmill
**Stamford 1978**	**A**	Arm cranking on a modified bicycle ergometer from a standing position
**Klausen 1974**	**A**	A bicycle ergometer adapted for arm exercise
**Clausen 1973**	**A**	A bicycle ergometer adapted for arm exercise

A, arm crank ergomter; W, wheelchair; H, handcycle
